# The plate body: 3D ultrastructure of a facultative organelle of alveolar epithelial type II cells involved in SP-A trafficking

**DOI:** 10.1007/s00418-020-01912-7

**Published:** 2020-09-02

**Authors:** Christian Mühlfeld, Christoph Wrede, Viktor Molnár, Alexandra Rajces, Christina Brandenberger

**Affiliations:** 1 Institute of Functional and Applied Anatomy, Hannover Medical School, Carl-Neuberg-Str. 1, 30625 Hannover, Germany; 2 Biomedical Research in Endstage and Obstructive Lung Research (BREATH), Member of the German Center for Lung Research (DZL), Hannover, Germany; 3 Research Core Unit Electron Microscopy, Hannover Medical School, 30625 Hannover, Germany; 4 Hanover Adventure Zoo, 30175 Hannover, Germany

**Keywords:** Surfactant protein A, Plate body, Alveolar epithelial type II cell, Electron tomography, Focused ion beam scanning electron microscopy, Immunocytochemistry

## Abstract

Plate bodies are facultative organelles occasionally described in the adult lungs of various species, including sheep and goat. They consist of multiple layers of plate-like cisterns with an electron dense middle bar. The present study was performed to elucidate the three-dimensional (3D) characteristics of this organelle and its presumed function in surfactant protein A (SP-A) biology. Archived material of four adult goat lungs and PFA-fixed lung samples of two adult sheep lungs were used for the morphological and immunocytochemical parts of this study, respectively. 3D imaging was performed by electron tomography and focused ion beam scanning electron microscopy (FIB-SEM). Immuno gold labeling was used to analyze whether plate bodies are positive for SP-A. Transmission electron microscopy revealed the presence of plate bodies in three of four goat lungs and in both sheep lungs. Electron tomography and FIB-SEM characterized the plate bodies as layers of two up to over ten layers of membranous cisterns with the characteristic electron dense middle bar. The membranes of the plates were in connection with the rough endoplasmic reticulum and showed vesicular inclusions in the middle of the plates and a vesicular network at the sides of the organelle. Immuno gold labeling revealed the presence of SP-A in the vesicular network of plate bodies but not in the characteristic plates themselves. In conclusion, the present study clearly proves the connection of plate bodies with the rough endoplasmic reticulum and the presence of a vesicular network as part of the organelle involved in SP-A trafficking.

## Introduction

Alveolar epithelial type II (AE2) cells are an essential part of the mammalian alveolar epithelium: they serve as progenitor cells of the terminally differentiated type I cells and produce the surface-tension lowering surfactant (Fehrenbach 2001). The latter is stored in and secreted from characteristic organelles, the so-called lamellar bodies (Weaver et al. 2002). AE2 cells are present in all terrestrial mammalian lungs ranging from the 2 g weighing Etruscan shrew to the largest terrestrial mammals, the elephant, with a body mass of several tons. Although differences exist among species concerning lamellar body ultrastructure or the number of AE2 cells per alveolus, the consistence of the AE2 cell morphology between various species is remarkable (Stone et al. 1992; Wirkes et al. 2010).

Nevertheless, there are species differences in AE2 cell ultrastructure which tend to be neglected if they are not present in the most widely used laboratory animals mouse and rat. One of these differences relates to an incidentally described organelle, in the beginning termed “bar-like structure” (Stephens et al. 1973), later addressed as “plate-like body” (Shibamoto et al. 1989) or “plate body” (Lakritz et al. 1992) or “highly ordered granule” (Miller et al. 2018). As the term “bar-like structure” was also used for a different type of organelle (Shimura et al. 1980), likely widened cisterns of the endoplasmic reticulum, we follow the recommendation of Shibamoto et al. (1989) to use the term “plate body” to address this organelle unambiguously. No matter what the name, this organelle was consistently described to be composed of a varying number of parallel, stacked membranous cisterns with a central electron dense line. The cisterns may differ in length and were described to be continuous with the rough endoplasmic reticulum but their membranes were devoid of ribosomes. Miller et al. (2018) reported that the side of the cisterns was covered with ribosomes though. Plate bodies have been reported in guinea pigs (Manabe and Ikeda 1986), ferrets (Miller et al. 1982), dogs (Miller et al. 1986), and sheep (Lakritz et al. 1992). Their appearance in dog and sheep lungs was described to be age-dependent with no plate bodies in newborn and increasing amount in older animals (Lakritz et al. 1992; Miller et al. 1986). Although various authors proposed a role in surfactant protein metabolism, particularly of surfactant protein A (SP-A) (Miller et al. 2018), the function of plate bodies is still unclear. A striking ultrastructural similarity of this organelle exists with Birbeck granules of Langerhans cells (Romani et al. 2010), in particular of Birbeck-like granules induced in cells of the M10 human melanoma cell line by expression of the protein langerin tagged with YFP (Lenormand et al. 2013). This might raise the possibility of plate bodies being involved in the immunological functions of AE2 cells.

Only little information exists with respect to the three dimensional (3D) ultrastructure of plate bodies. However, ultrastructural knowledge may help to provide new hypotheses on the function of this organelle. Therefore, in the present study we used archived material from adult goat lungs in which plate bodies had been identified by transmission electron microscopy. Two high-resolution methods of 3D analysis were used to better characterize the ultrastructure of plate bodies, namely electron tomography and focused ion beam scanning electron microscopy (FIB-SEM). In addition, to further elucidate the function of plate bodies, lung samples of adult sheep were investigated by immuno electron microscopy to identify whether plate bodies are involved in the biology of SP-A.

## Material and methods

### Sample origin and preparation

Archived material of four African pygmy goats (*Capra aegagrus hircus*) was generously provided by Ewald R. Weibel, University of Bern, Switzerland. The animal experiments and the processing of the samples were described previously (Weibel et al. 1987) and used for 3D visualization of plate bodies. The origin of the specimens was the Concord Field Station in Harvard, Boston, USA. The lungs were intratracheally fixated with 2.5% glutaraldehyde in potassium phosphate buffer (pH 7.4) and sampled with systematic uniform random sampling. Tissue blocks were post-fixated and stained with OsO4 in sodium cacodylate buffer and uranyl acetate in maleate buffer, respectively. After a series of incubation and washing steps with ascending ethanol concentrations, the samples were embedded in epoxy resin.

Lung samples of two Cameroon sheep (*Ovis aries aries cameroon*) for immuno gold labeling were provided by the Hanover Adventure Zoo. The sheep were slaughtered within the legal framework of the European Union for zoo animals (Animal by-products Regulation No 1069/2009 of the European Parliament and of the Council) for the use of feeding other zoo animals by penetrating captive bolt stunnings and subsequent exsanguination. During post-mortem carcass examination and meat inspection, small samples (appr. 0.5 cm × 0.5 cm × 0.5 cm) were taken from various regions of the lung and fixed by immersion in 4% paraformaldehyde buffered with 0.2 M Hepes (pH 7.35). Samples for immuno gold labeling were embedded via cryo-substitution in Lowicryl HM20 (Fa. Polysciences, # 15,924) as described previously (Mühlfeld and Richter 2006).

### Serial TEM tomography

Epoxy resin embedded samples were cut in series of 300 nm thick sections and mounted onto mesh copper grids. The samples were screened for suitable areas, containing a plate body in several consecutive sections with a Morgagni 268 TEM (FEI, Netherlands). Serial tomography imaging was recorded on selected areas with a Tecnai F20 TEM (FEI) equipped with a 4 K Eagle-camera (FEI) at a voltage of 200 kV. The tomography was recorded at a magnification of 11,500x, performing a continuous tilt angle shift from max − 69° to + 69° with an increment of 1°. Prior to tomography assessment, ultrathin sections were coated with 10 nm colloidal gold beads (Plano GmbH, Germany) as fiducial markers for 3D stack reconstruction. Image processing and 3D stack reconstruction were performed with etomo [part of the iMOD package (Kremer et al. 1996)]. Further image processing and alignment of four consecutive image stacks were performed with Fiji (Schindelin et al. 2012) resulting in an image stack with 341 images. 3D reconstruction and visualization of plate bodies was done with 3dmod [part of the iMOD package (Kremer et al. 1996)].

### FIB-SEM

Ultrathin sections (60 nm) were cut from the resin embedded samples and inspected in a TEM (Morgagni 268, FEI) to locate regions of interest for FIB-SEM analysis. The trimmed resin block was clamped in a slotted SEM specimen holder and the sides were varnished with conductive silver (Plano GmbH). After sputter coating (20 nm gold; Quorum Q150R ES sputter coater; Quorum Technologies Ltd, United Kingdom), the target region was identified in a Zeiss Crossbeam 540 including the ATLAS software package (Carl Zeiss Microscopy GmbH, Germany). A deposition of platinum was used to protect the sample surface and marks were milled and highlighted with carbon for tracking, autofocus and autostigmation correction. The region of interest was recorded [1831 images; approximately 32 µm (*x*) × 15 µm (*y*) × 18.31 µm (*z*)] with the Inlens Secondary Electron and the Energy selective Backscattered detector (grid voltage 800 V) at 1.5 kV acceleration voltage and 1.0 nA beam current with a pixel size of 2 nm and a section thickness of 10 nm. A substack of 1088 images was converted to 8 bit and cropped in Fiji (Schindelin et al. 2012). 3D reconstruction and visualization of the plate body was done with 3dmod [part of the iMOD package (Kremer et al. 1996)].

### Immuno gold labeling

Ultrathin sections of Lowicryl embedded samples were cut and placed on nickel grids for immuno gold labeling with anti-SP-A antibody as previously explained in detail (Schmiedl et al. 2005). The primary anti-SP-A antibody (Merck, AB 3420-I, rabbit anti-human SP-A) was used at a dilution of 1:1000 and the secondary antibody (goat anti-rabbit 10 nm gold, Plano EM, # 17010-1) at a dilution of 1:50. Images were recorded with a Morgagni 268 microscope (FEI). No primary controls were done to test the specificity of the secondary antibody labeling.

### Western blot

Western blot to test SP-A labelling ability on sheep lung tissue was done with 4% PFA fixated tissue. Protein isolation was conducted with the Qproteome FFPE Kit (Qiagen), particularly optimized to isolate protein from PFA fixed tissue. Western blot was done as previously described (Hollenbach et al. 2019). Samples from two different sheep lungs were used (sample #1 and #2) and loaded on the gel in two different volumes (2.5 µL and 5 µL). The primary antibody was diluted at a concentration of 1:1000 and the secondary (goat anti-rabbit, Dianova # 111-036-144) at a concentration of 1:2000.

## Results

**Fig. 1:**
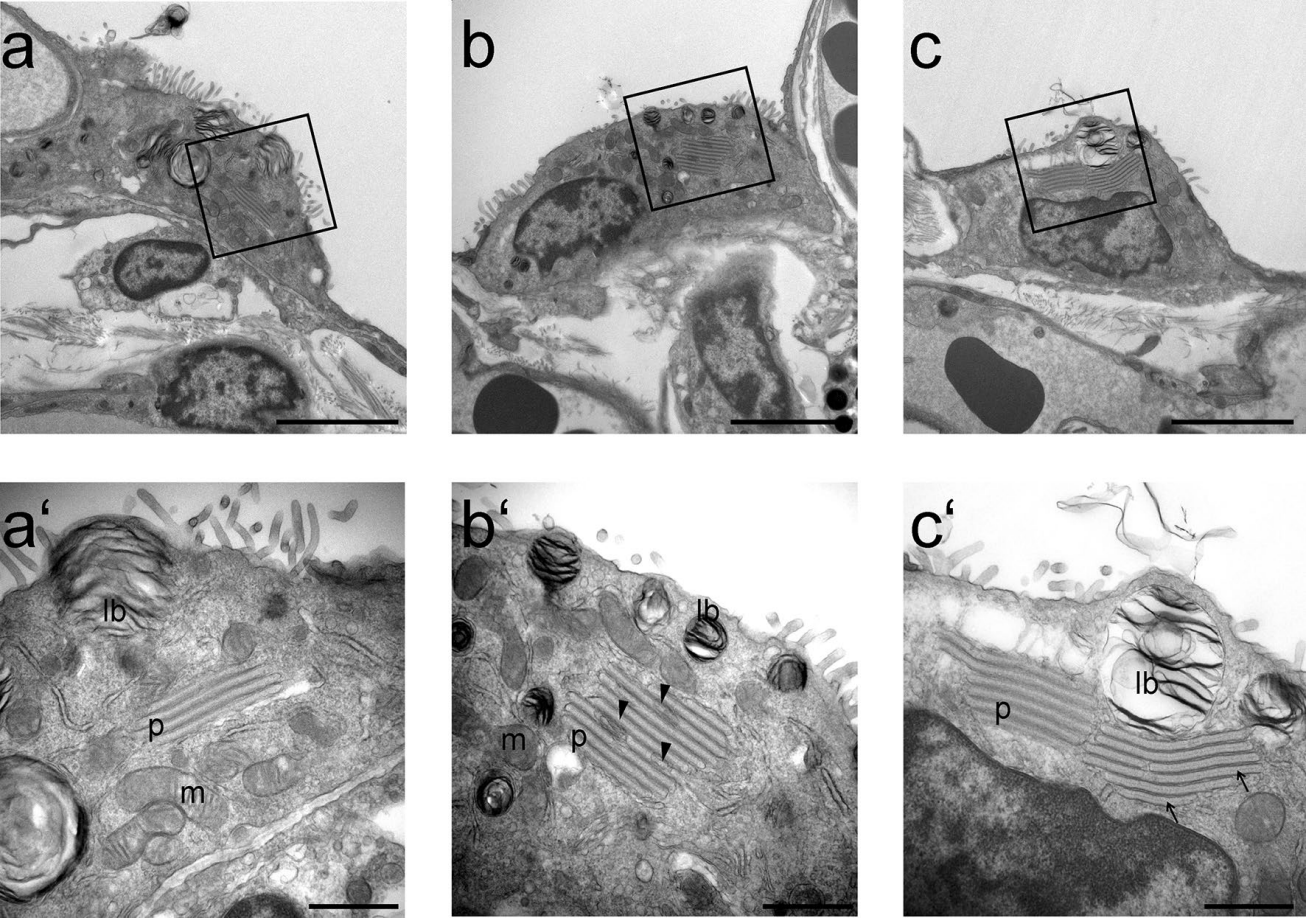
TEM images of plate bodies of African pygmy goats. The number of plate-like cisterns in alveolar type II cells was varying from 2–4 plates (figure a/a´) up to multiple connected stacks with 6–10 cisterns (figure b/b’ and c/c’). The plate bodies showed a characteristic electron dense median line present in the plate-like cistern (arrow). Some of the cisterns also show some vesicular inclusion (arrow heads); p = plate body, lb = lamellar body, m = mitochondria. Scale bar (a, b, c) = 4 µm, scale bar (a’, b’, c’) = 1 µm

The plate body, consisting of a series of plate-like cisterns has been reported as an organelle present in AE2 cells of different species. In this study, the plate body was frequently found in AE2 cells of lung samples of the African pygmy goat and the Cameroon sheep. All 3D EM analyses were performed on goat lung samples, whereas immuno labeling was done on sheep lung samples. Using transmission electron microscopy (TEM), the plate-like cisterns of the plate body were detected in almost every third or fourth cell in different size and formation (Fig. 1). The number of plate-like cisterns varied between 2 and 4 plates (Fig. 1a) up to multiple connected stacks with 6–10 cisterns (Fig. 1b, c). The height of the individual cisterns was constantly around 100 nm, while the length of the cisterns varied. Additionally, the plate bodies showed a characteristic electron dense median line present in the plate-like cisterns. Some of the cisterns also showed vesicular inclusions or vesicles at the end of the cisterns (Fig. 1b).

TEM tomography and FIB-SEM in combination with image reconstruction were used to visualize the 3D ultrastructure of the plate bodies. While TEM tomography allows a high resolution and detailed view of the 3D structure, FIB-SEM enables an overview reconstruction of the cell and its content and allows displaying a plate body as a whole.

**Fig. 2:**
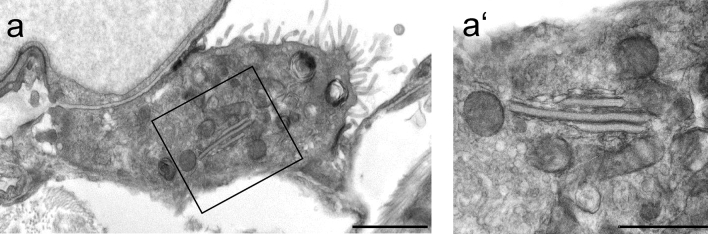
TEM image of plate body containing alveolar type II cell selected for TEM tomography (African pygmy goat). The plate body containing cell, shown in figure a (scale bar = 2 µm) and at higher magnification in a’ (scale bar = 1 µm), was present in four consecutive sections which were further imaged by TEM tomography for plate body reconstruction

**Fig. 3:**
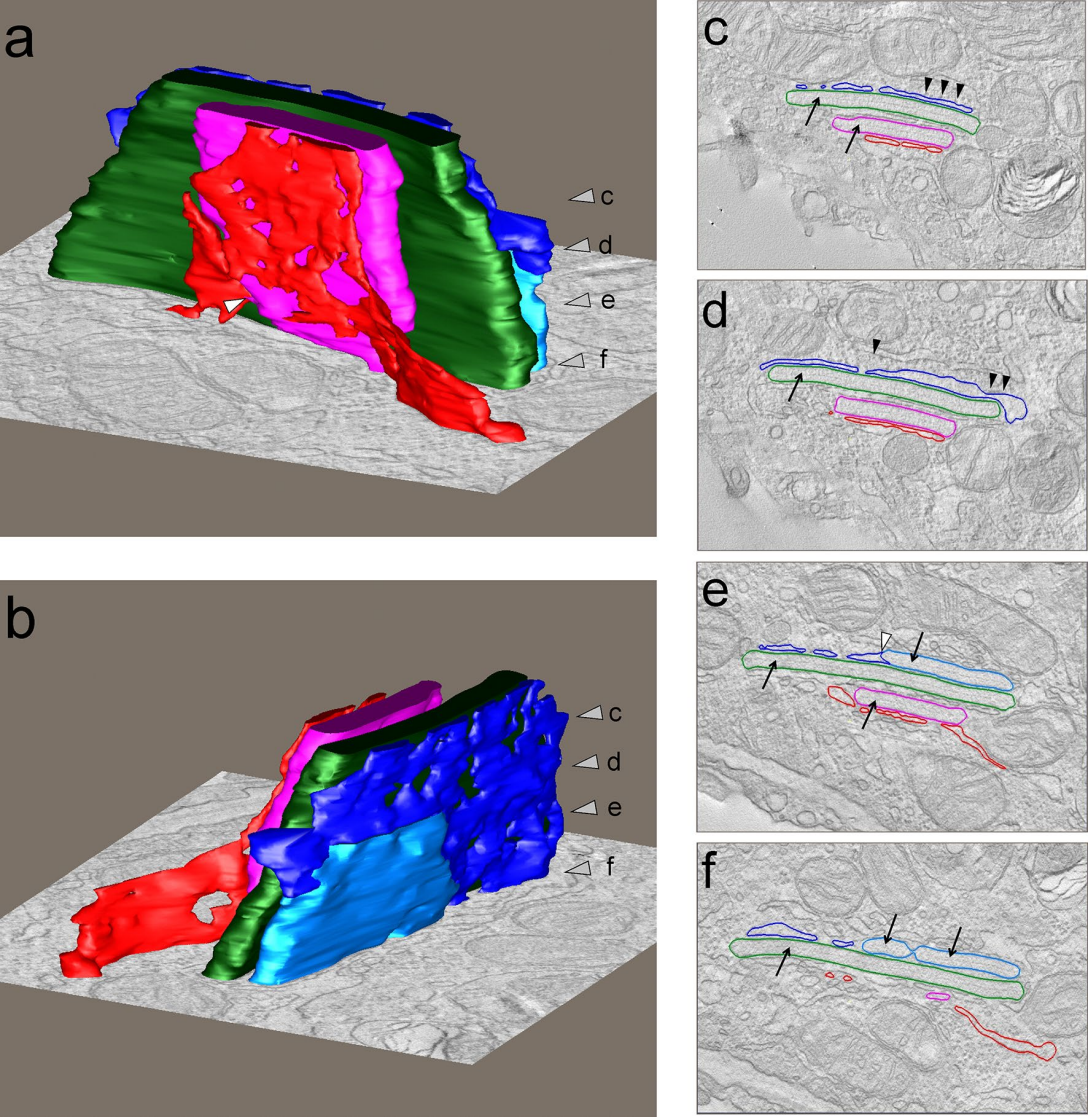
Reconstructed TEM tomogram and model view of plate bodies (African pygmy goat). Plate bodies were reconstructed by surface rendering of the plate body membranes and 3D view with three different cisterns in pink, green and light blue is shown in figures a and b. The grey arrow heads in figure a and b represent the virtual planes shown in figures c, d, e and f. The light blue cistern is in connection (white arrow head in figure e) with a vesicular network (dark blue), belonging to the ribosome containing rER (black arrow heads figure c and d). The red vesicular network is also in direct connection with the cistern displayed in pink (see white arrow head figure a). The electron dense midline (arrows figures c, d, e and f) is only apparent in the regularly formed cistern but not in the vesicular network

For TEM tomography serial sections with an approximate thickness of 300 nm were generated and analyzed for plate body containing AE2 cells. A plate body containing cell, shown in Fig. 2, was present in four consecutive sections and selected for TEM tomography. The reconstructed TEM tomogram is shown in Fig. 3. Three plate-like cisterns were present here and are displayed in pink, green and light blue. The light blue outer cistern transforms into a vesicular network (displayed in dark blue) that shows features of ribosome-containing rough endoplasmic reticulum. The other outer cistern, displayed in pink, is also in direct connection with a vesicular network that resembles rough endoplasmic reticulum. Only the middle, green cistern is not connected to another structure within this tomogram section. It becomes furthermore apparent, that the characteristic electron dense midline is only present in the regularly formed cisterns, but not in the vesicular networks.

**Fig. 4:**
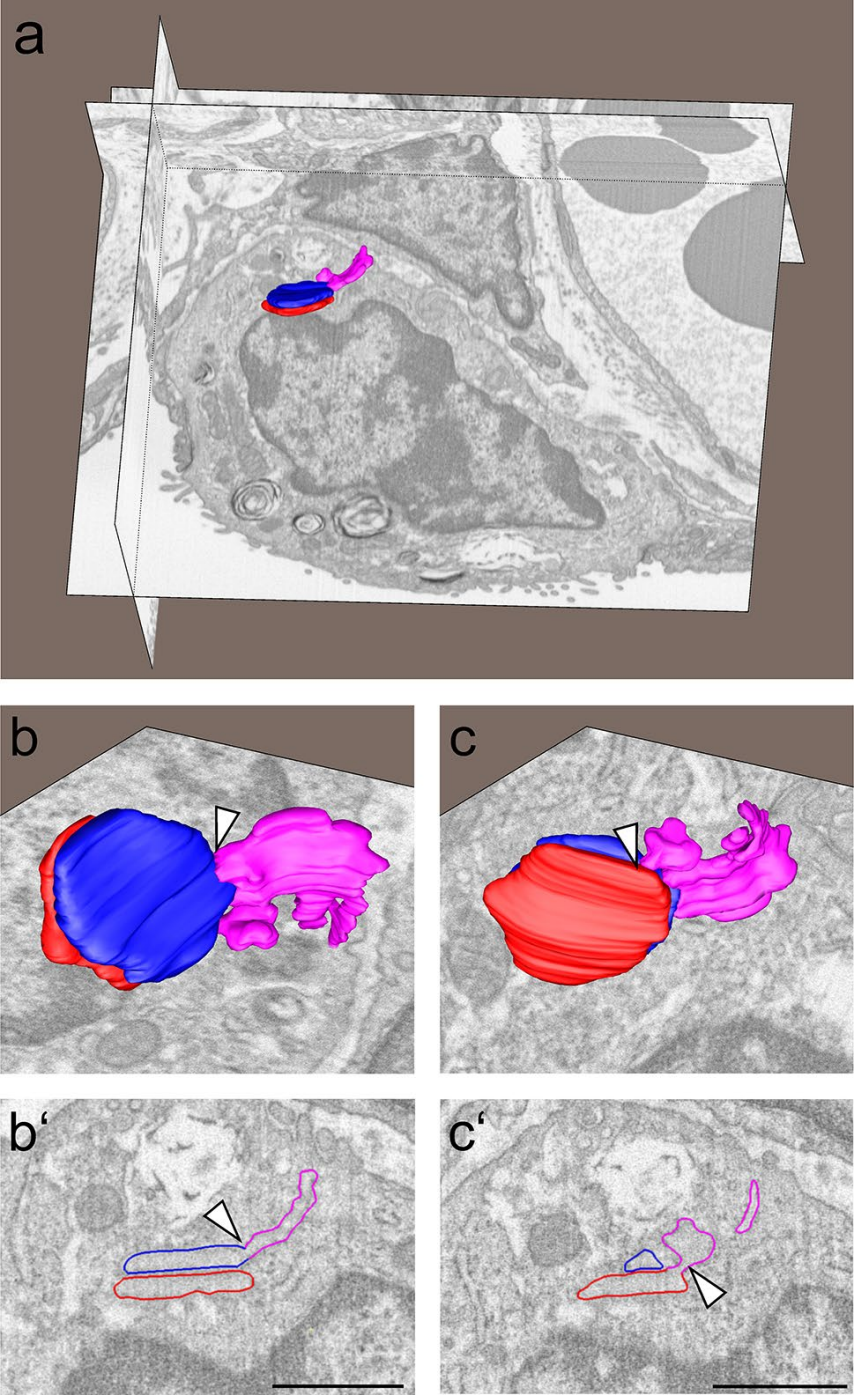
FIB-SEM image stack and 3D reconstruction on an entire plate body (African pygmy goat). An overview of an alveolar type II cell with a plate body is shown in figure a. The two plate-like cisterns, shown in blue and red, are connected with each other by a vesicular network shown in pink. The vesicular network shows features of smooth and rough ER. The connection of the blue with the network is indicated by an arrow head in figure b and b’ and the connection between the red cistern in figure c and c’. Scale bar = 1 µm

FIB-SEM imaging of an AE2 cell containing an entire plate body was further performed. The 3D image reconstruction of the plate body is shown in Fig. 4. The imaged plate body only contains two cisterns and is therefore relatively small compared to other stacks with up to ten cisterns. The two plate-like cisterns are connected with each other by a vesicular network. The vesicular network shows features of smooth and rough endoplasmic reticulum, similarly as shown in Fig. 3 by TEM tomography. The reconstruction provides evidence that the cisterns are connected at several levels and most likely form an interconnected and potentially dynamic organelle. Here, as well as in other images, the plate body was found in close proximity to the nucleus indicating its relationship to the endoplasmic reticulum, but not as part of the perinuclear envelope.

**Fig. 5:**
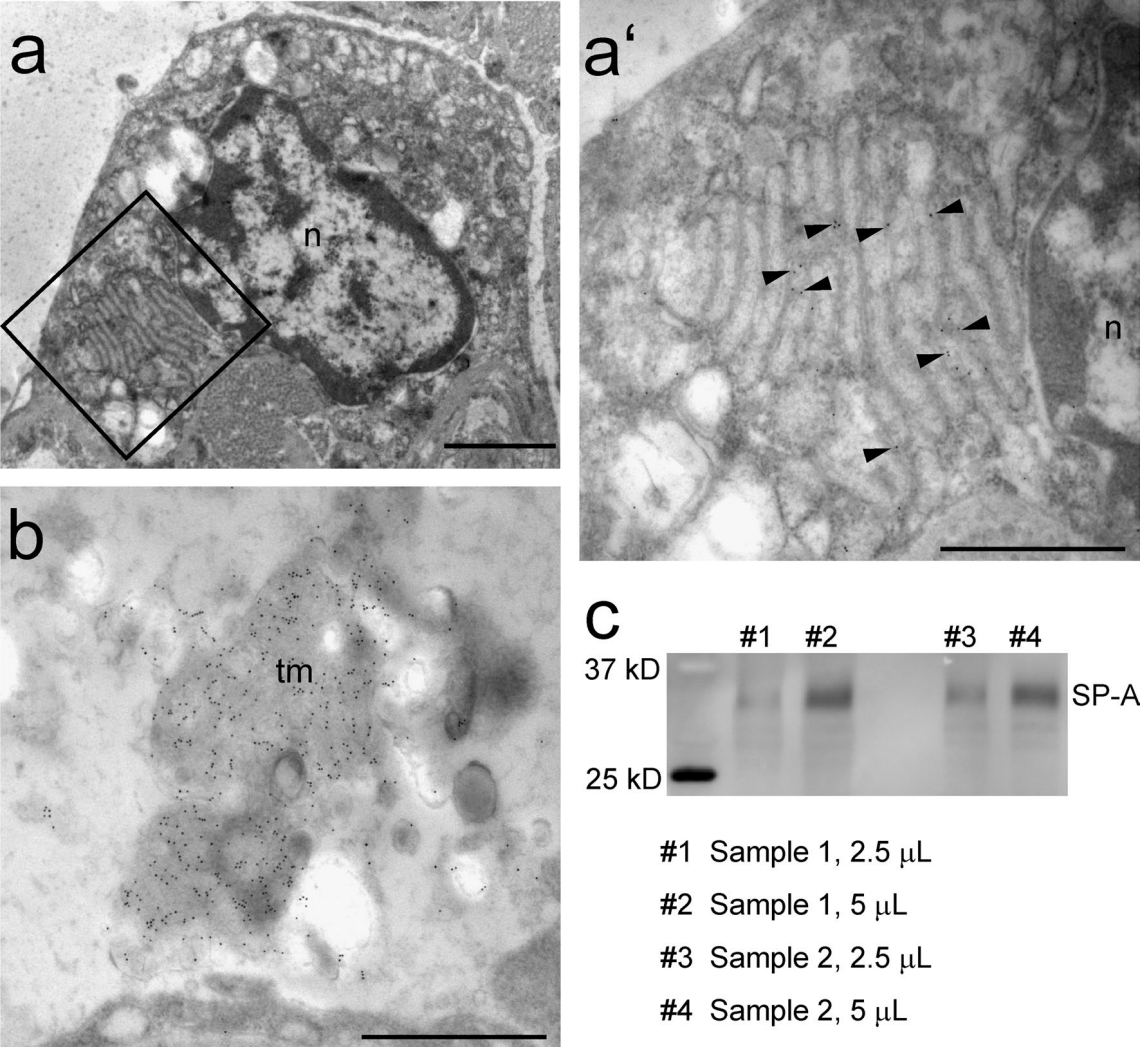
Immuno gold labeling against surfactant protein A (Cameroon sheep). **a** A large plate body is visible in an alveolar type II cell of a Cameroon sheep. Scale bar = 2 µm. **a’** Higher magnification of the boxed area in a demonstrating the immuno gold labeling for SP-A (arrow heads) at the vesicular regions of the plate body. n = nucleus, scale bar = 1 µm. **b** Strong immuno labeling for SP-A was observed over the intra-alveolar surfactant subtype tubular myelin (tm). Scale bar = 1 µm. **c** Western blot of PFA-fixed Cameroon sheep lung tissue showing a clear band at 35 kDa

Figure 5a shows an over view of an AE2 cell with a prominent plate body consisting of a stack of approximately 12 cisterns with a high number of intercisternal vesicles or vesicular inclusions. At higher magnification (Fig. 5a’), a clear immuno gold labeling for SP-A was visible at the sites of the vesicular inclusions of the plate body, but neither at the outer membranes nor the electron dense bar within the plate-like cisterns. Although the labeling of plate bodies was relatively weak in contrast to that of tubular myelin, a lattice-like subform of intra-alveolar, secreted surfactant (Fig. 5b), it could be clearly distinguished from the low background labeling. Hardly any gold particles were found in no primary controls.

Although the antibody against SP-A has been successfully used by other authors, it has not been used in sheep lungs so far. Therefore, to test the specificity of the antibody in this species, Western blot was performed on the PFA-fixed samples of the Cameroon sheep. Both sheep lungs showed a clear single band at about 35 kDa, the molecular weight of the SP-A monomer chain (Fig. 5c).

## Discussion

The present study confirms the presence of a highly structured organelle consisting of various numbers of membranous cisterns with an electron dense middle bar in the adult goat and sheep lung. The ultrastructure of this organelle is consistent with previous reports (Stephens et al. 1973; Shibamoto et al. 1989). Due to their 3D characteristics, the individual cisterns had an appearance of plates or discs, the whole organelle reminded of a stack of plates, hence the name plate body. 3D visualization also confirmed that plate bodies were continuous with the cisterns of the rough endoplasmic reticulum, and did only occasionally possess ribosomes at the surface of their vesicular network, mainly located at the sides of the plates. This vesicular network may be layered between or on the outer surface of plate bodies, thus forming “perforated plates” or at the sides of the organelle. Immuno electron microscopy showed a clear gold labeling for SP-A at the vesicular network within or at the sides of the cisterns.

Previous studies have mainly used single or serial sections (TEM) to analyze the ultrastructure of plate bodies. Here, we have employed new techniques that allow a 3D visualization of the plate bodies at high resolution. Electron tomography is based on imaging of thick sections (300–500 nm) at defined tilting angles and back-projection of the resulting images to provide a stack of parallel images. The advantage of this technique is the high resolution in all three dimensions. Disadvantages relate to the missing wedge (due to high tilting angles) and the small volume that can be analyzed (Baumeister et al. 1999; McEwen and Frank 2001; Ochs et al. 2016). To increase the volume, the tomographic visualization shown in this manuscript is based on tomograms of four consecutive 300 nm thick sections. A relatively new method is FIB-SEM which has only rarely been used in lungs so far (Købler et al. 2014; Ochs et al. 2016; Hegermann et al. 2019; Schneider et al. 2020). The *x*–*y* resolution does not reach the levels of TEM tomography but allows visualization of larger volumes (whole cells) with a *z* resolution of approximately 10 nm. For the sake of completeness, a third method of volume electron microscopy, serial block-face SEM, should be mentioned which offers less resolution than FIB-SEM in all dimensions but allows visualization of larger volumes of the lung such as multiple mouse alveoli (Buchacker et al. 2019) or parts of a human alveolus (Schneider et al. 2019).

The number of studies about plate bodies is relatively limited, a fact that is partly explained by their absence in humans as well as mice and rats. The absence of plate bodies in previous studies of human lungs lowers the interest of a medicine-oriented life science community although knowledge about the function of this organelle might improve our general understanding of AE2 cell biology. The absence in mice and rats also complicates the situation because the availability of genetically modified animals, fresh lung samples and the suitability of antibodies is limited.

Thus, the function of plate bodies is still under debate. Under normal conditions, plate bodies do not seem to be essential for AE2 cell biology as they are not present in all AE2 cells from one animal (which, however, may be a false interpretation due to 2D imaging), they do not occur in all animals of the same species and they have not been reported in newborn animals of species known to contain plate bodies so far. Their contribution to surfactant metabolism, particularly surfactant proteins, has been proposed by several authors (Stephens et al. 1973; Manabe and Ikeda 1986; Miller et al. 2018) and has been challenged by others (Shibamoto et al. 1989). Plate bodies lack a clear connection with lamellar bodies or multi-vesicular bodies which raised the doubts about their importance for surfactant metabolism. The increase of plate bodies during life (Miller et al. 1986), however, raises the possibility that plate bodies occur as a reaction to environmental conditions, such as inhalation of certain patho-/antigens or toxicants or exposure to certain nutrients. The original work of Stephens et al. (1973) showed an increase of plate bodies as a reaction to ozone exposure. As such, plate bodies might also be related to the immune system of the animals. The similarity of plate bodies with Birbeck granules of Langerhans cells is supporting this hypothesis. Birbeck granules are also composed of plates of cisterns and an electron dense middle bar which contains the protein langerin, a collectin involved in pathogen binding, endocytosis and Birbeck granule formation (Valladeau et al. 2000; Feinberg et al. 2010).

However, a contribution of plate bodies to either surfactant protein metabolism or to an immune function is not a contradiction as surfactant plays a role in the innate immune defense of the lung. Surfactant is an essential component of healthy mammalian lungs consisting of 90% lipids (mainly phospholipids) and 10% proteins, among these the surfactant proteins A, B, C and D (SP-A, SP-B, SP-C and SP-D) (Lopez-Rodriguez et al. 2017). The main function of surfactant is the lowering of the surface-tension at the air–liquid interface at the surface of the alveolar epithelium. Among the surfactant proteins, in particular, SP-B and SP-C are involved in the surface-lowering properties of surfactant with SP-B being an absolutely essential component (Brasch et al. 2004). While SP-A and SP-D knockout mice also show alterations of the surfactant system (Ikegami et al. 1997; Botas et al. 1998), these hydrophilic proteins are also known to be important for host defense (Lawson and Reid 2000). SP-A is a C-type lectin with a collagen-like domain, hence belongs to the collectin family of proteins. Mature SP-A consists of six subunits each of which is a trimer of the 35 kDa SP-A monomeric polypeptide chain (Vieira et al. 2017). In the current study, the Western blot band was found at this molecular weight although other bands are known from the literature, for example the trimeric band at 105 kDa (Vieira et al. 2017). Over the years, various immune functions of SP-A (and SP-D) have been described, among these, binding and opsonization of viruses, bacteria, worms, allergens, and apoptotic cells, increasing phagocytosis and modulation of cytokine and inflammatory mediator expression (Watson et al. 2019). Thus, if plate bodies were involved in the metabolism of SP-A, this, in turn, would mean that plate bodies are involved in the innate host defense of the lung.

The hypothesis of the involvement of plate bodies in surfactant metabolism was previously based on morphological interpretation. The present study used an immuno gold labeling for SP-A to test this hypothesis. It provides clear evidence that plate bodies possess a connection to SP-A, probably its synthesis or packing in vesicles. It contrasts with the morphological interpretation of Miller et al. (2018) because the gold particles were not found in the plate-like cisterns themselves, but rather at the vesicular periphery of the cisterns. In the human lung, intracellular SP-A was shown to be localized in vesicles or multivesicular bodies of AE2 cells, only rarely at the outer membrane of the phospholipid-storing lamellar bodies, indicating an alternative pathway not related to lamellar body secretion (Ochs et al. 2002). Here, we showed that besides the strong and clear labeling of tubular myelin, the main gold labeling of SP-A was observed at the vesicular regions of plate bodies suggesting that this organelle may have a similar function in SP-A trafficking as multivesicular bodies and may serve as a structural variant.

In conclusion, plate bodies are a facultative organelle of AE2 cells in the lungs of adult sheep and goats (among other) which consist of a varying number of plate-like cisterns and a vesicular network involved in SP-A trafficking.

## Data Availability

Not applicable.
